# An Epsilon‐Near‐Zero‐Based Nonlinear Platform for Ultrafast Re‐Writable Holography

**DOI:** 10.1002/nap2.70016

**Published:** 2026-01-25

**Authors:** M. Zahirul Alam, Robert Fickler, Yiyu Zhou, Enno Giese, Jeremy Upham, Robert W. Boyd

**Affiliations:** ^1^ Department of Physics University of Ottawa Ottawa Ontario Canada; ^2^ Photonics Laboratory, Physics Unit Tampere University Tampere Finland; ^3^ Institute of Optics University of Rochester Rochester New York USA; ^4^ Department of Electrical Engineering Yale University New Haven Connecticut USA; ^5^ Fachbereich Physik Institut für Angewandte Physik Technische Universität Darmstadt Darmstadt Germany

## Abstract

We re‐examine real‐time holography for all‐optical structuring of light and optical computation using a contemporary material: a subwavelength‐thick, spatially unstructured film of indium tin oxide (ITO). When excited by spatially structured light at epsilon‐near‐zero frequencies, the film acts as an efficient and reconfigurable diffractive optical platform for all‐optical modulation of light such as spatial structuring and optical computations. We demonstrate a few percent of absolute diffraction efficiency over greater than 300‐nm‐bandwidth around telecom wavelengths using a film four orders of magnitude thinner than and up to six orders of magnitude faster than standard holographic materials. Our findings highlight the potential of using epsilon‐near‐zero‐based nanostructures for efficient modulation of spatially structured light and rapid prototyping without complex nanofabrication processes.

## Graphical abstract

We show that an unstructured, sub‐wavelength thick epsilon‐near‐zero material, such as ITO, can be efficiently used for ultrafast re‐writable holography over a broad bandwidth range. We showcase two powerful applications of our scheme, that is, spatial structuring of light and optical computation.

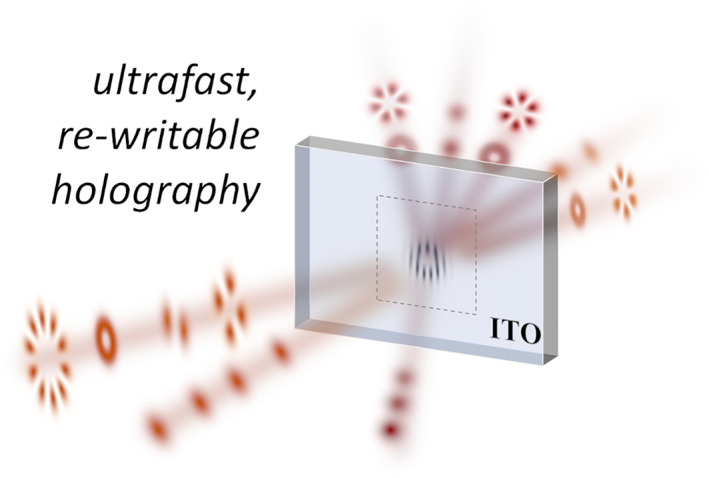

## 1 Introduction

The unmatched speed and parallelism of light offers tremendous opportunities to address the energy‐density, heat, and bandwidth constraints of electrical interconnects in a computing platform [1]. The interest in using optical systems for information processing is not new, as even a simple lens can passively perform parallel Fourier transforms [2]. Optical processing technologies have been widely investigated throughout the late twentieth century [3, 4, 5], such as the use of holographic techniques and nonlinear wave mixing in Fourier space for convolution and correlation operations [6, 7, 8, 9]. Recent advances in metamaterials [10] and metasurfaces [11, 12], have led researchers to leverage wave physics for novel approaches to information processing [13, 14, 15, 16, 17, 18] for applications in hardware acceleration [19] and machine learning [20, 21, 22, 23]. These demonstrations of optical processing using diffractive optics and metasurfaces rely largely on nanofabricated or 3D‐printed static structures [24]. Such structures are written once and then used as a fixed layer for image processing or classification tasks with limited tunability (on/off), for example, through temperature [25] or mechanical deformation [26]. These demonstrations highlight the massive parallelization potential of free‐space diffractive optical computing structures and metasurfaces by exploiting spatial modes, wavelengths, time, polarizations, spins, and the orbital angular momentum of light. However, one of the challenges in this aspect is to implement full dynamic reconfigurability and on‐demand tunability of diffractive optical surfaces [12, 24].

In this work, we implement a real‐time holographic protocol by exploiting the properties of topologically structured light fields [27, 28] and the sub‐picosecond, unity‐order nonlinear optical response of a transparent conductive oxide at its epsilon near‐zero (ENZ) frequencies [29, 30, 31]. We experimentally demonstrate, using an unpatterned, thin ENZ film, a rapidly reconfigurable computing surface capable of ultrafast copying of spatial modes of light, broad‐band operation over more than 300 nm, and all‐optical first‐order differentiation.

Traditional real‐time holographic materials, such as photo‐refractive crystals [32, 33] and photo‐polymers [34, 35, 36], have been extensively studied for all‐optical computation. However, these materials suffer from slow refresh rates (milliseconds to seconds) [34], low index contrast, are typically several millimeters thick, and sometimes require the read and object beams to be of widely different frequencies, for example, deep‐UV for writing and visible for reading [32, 37]. In contrast to traditional holographic materials, when indium tin oxide (ITO)—a degenerately doped transparent conducting oxide widely used in touch screens and photovoltaics—is excited at its ENZ frequencies, it exhibits superior performance: ITO can exhibit unity‐order index variations for high‐contrast diffraction patterns with sub‐picosecond refresh times, and allows for operation using infrared light in both frequency‐degenerate and frequency‐non‐degenerate configurations. These findings suggest that ITO‐based surfaces could enable a wide range of applications, including rapid prototyping without complex nanofabrication processes [38].

## 2 Transferring Information Between Optical Pulses

The general scheme of our experiment is as follows. A Gaussian reference beam is brought to interfere with a frequency‐degenerate object beam carrying an arbitrary image in the Fourier plane. All beams are ∼120‐fs laser pulses and have a beam diameter of around 50–90 μm at the plane of the ITO depending on the modal characteristics. A 310‐nm‐thick, transparent ITO film on a glass substrate is placed on the same Fourier plane. Due to the intensity‐dependent response of the ITO's refractive index, the interference pattern induces a transient, spatially‐varying, intensity‐dependent refractive index distribution with high contrast between the unchanged (at the dark fringes) and nonlinearly changed index (at the bright fringes) [29]. The induced transient metastructure, that is, the hologram encoded using this technique carries the full spatial amplitude and phase information of the object beam. Furthermore, the encoding time of the interference pattern in the ITO thin film is less than 200 femtoseconds and it completely returns to the initial, uniform index within 1 picosecond. Consequently, a thin layer of ITO acts as an ultrafast, re‐programmable diffractive metasurface—programmed by the spatial patterns of the interfering structured light fields. Here we demonstrate such reconfigurability through two instructive applications, namely transduction of a spatial phase and intensity pattern to a read beam of varying wavelengths and edge‐detection of an image using first‐order differentiation.

**FIGURE 1:**
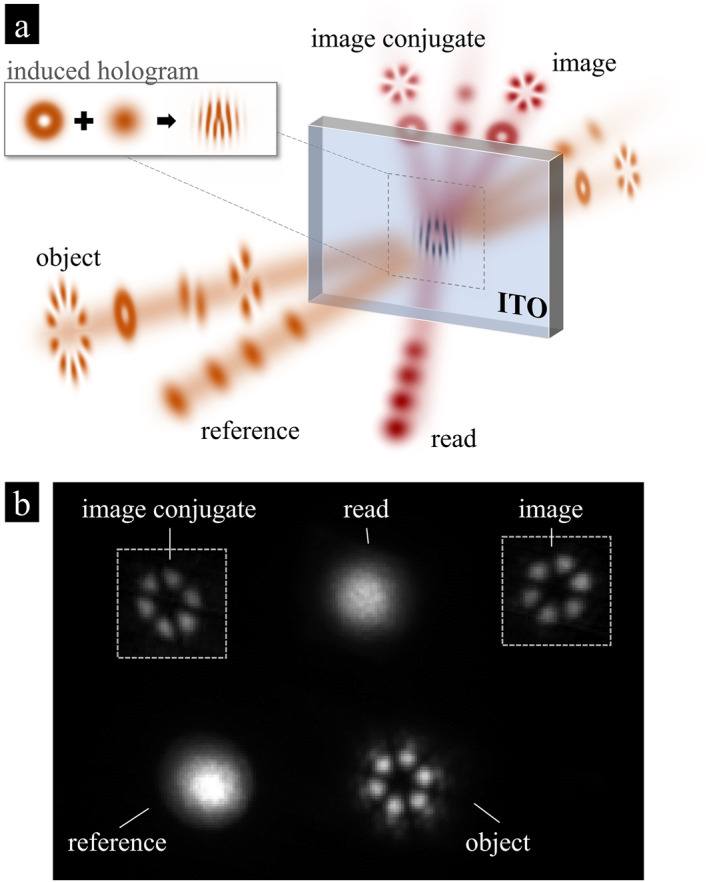
Experimental configuration. (a) Interference of the object and the reference beams (orange) imprint a spatially varying hologram (a transient metastructure) onto the ITO film through an intensity‐dependent change of refractive index (the example shown in the inset is for an object beam carrying one unit of OAM, i.e., ℓ=1). A read beam (red) of a distinct wavelength diffracts off the hologram. The diffracted image (conjugate image) beam carries the same (complex conjugate) spatial structure as the original object beam. (b) A camera image taken of the beams exiting the ITO film shows all relevant beams. For clarity, the two image beams were brightened by a factor of 10 (adjusted area indicated by dashed line). See Supporting Information S1: Figure S2 for the original unprocessed image.

In Figure 1a we display a schematic representation of the holographic recording and image transduction processes using the induced hologram (shown in the inset) formed by the object and reference beams of wavelengths near the zero‐permittivity wavelength of ITO, that is, 1260 nm. To read out the induced hologram, we illuminate it with a read beam of the same wavelength but low power to operate in the linear regime with negligible influence on the induced hologram. The resulting linear diffraction of the read beam results in two distinct copies of the initial object: the image and its conjugate counterpart. The image represents a reconstruction of the original object, whereas the counterpart is a complex conjugate image of the object. In practice, we placed a white screen in the far‐field after the ITO and used an infrared camera to image the screen (see Figure S1 in Supporting Information S1 for more details on the setup). Figure 1b is an example recording that captures all five beams, demonstrating that the intensity pattern of the object beam has been copied into the diffracted read beams (i.e., the image beam).

**FIGURE 2:**
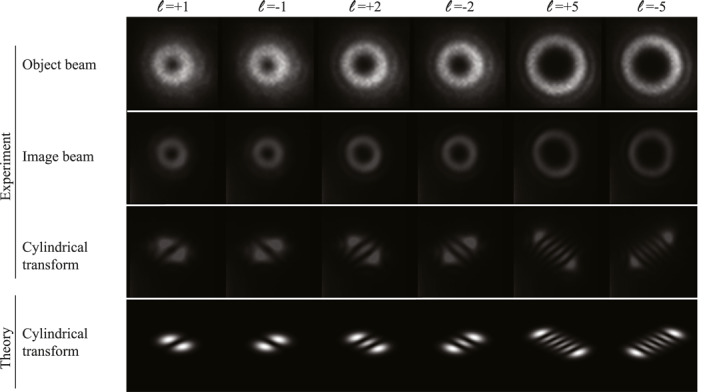
Transfer of spatial amplitude and phase information. The induced holograms are sensitive to the spatial amplitude and phase of the object beams. To confirm the transfer of phase information, we prepare object beams with different signs and values of OAM (ℓ=±1,2,5) and show the spatial structure of the resulting image beams. By performing cylindrical transformations and comparing the spatial structure and orientation of the transformed image beams with theory, we confirm the transfer of spatial phase information.

In addition to the intensity, we investigate the transfer of the spatial phase information to the read beam. We encode different orbital angular momentum (OAM)‐carrying structures onto the object beam by shaping the light's transverse phase using a spatial light modulator. OAM‐carrying light fields have an azimuthal phase gradient about the optical axis from 0 to 2πℓ, where ℓ corresponds to its integer OAM value [39]. The twisted phase structure leads to a phase singularity and thus a vanishing intensity along the optical axis, resulting in a donut‐like intensity pattern with a radius that increases with the OAM value (Figure 2, first row for different experimental recordings of the object beam). When detecting the resulting intensity patterns of the image beams (Figure 2, second row), we find that their intensity shapes match the corresponding original object beams, that is, they show the same donut‐shaped intensity structure that increases in radius with ℓ. We verify the correct spatial phase distribution of the image beams by performing an astigmatic mode conversion using a cylindrical lens [40]. The mode conversion process converts OAM beams into Hermite–Gauss (HG) modes that have a number of intensity lobes equal to |ℓ|+1, arranged in a line oriented according to the sign of ℓ [41]. Comparing the intensity patterns obtained after this cylindrical transform (Figure 2, third row) with the theoretical expectation (Figure 2, fourth row), we find very good agreement, verifying that we have transferred the amplitude and phase information of the light field from the object beam to the image beam.

## 3 Quantifying Transfer Efficiency

**FIGURE 3:**
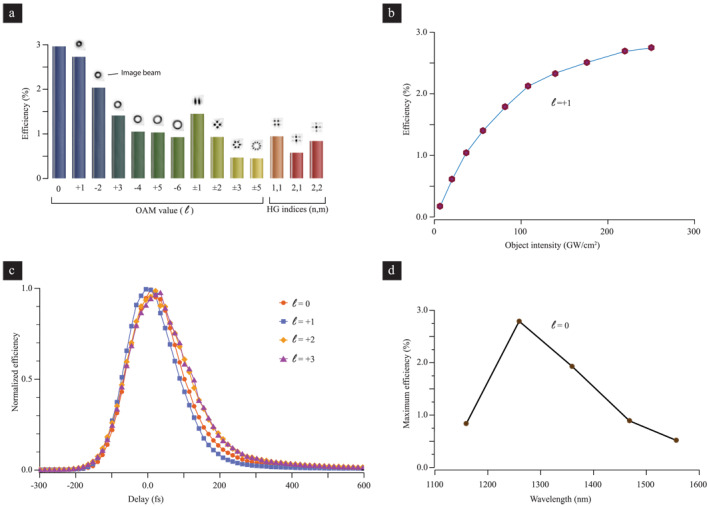
Efficiency of the information transfer process. (a) The maximum efficiencies of various OAM modes, their superpositions, and a few Hermite‐Gaussian modes. (b) Dependence of information transfer efficiency on object beam intensity with ℓ=+1. We set the intensity of the reference beam to be $300\;GW/cm^{2}$ and measure the diffraction efficiency of the image beam as a function of the object beam intensity at the ITO layer. (c) Temporal duration of the induced transient holographic structure. We vary the delay between the induced holographic structures (formed by the reference and object beams) and the read beam while recording the diffraction efficiency of the generated image beam for various spatial modes. We find that the hologram is only induced for the duration of a few hundred femtoseconds, suggesting a refresh rate of up to 1THz. (d) Tolerance of read beam wavelength. Although the object and reference beams were set to 1260 nm, we varied the read beam wavelength, showing significant image diffraction efficiency across more than 300 nm.

These initial experiments demonstrate the potential of ITO for applications in real‐time holography. Next, we investigate the efficiency of the transfer process by measuring the diffracted power carried by the image beam. We measure this efficiency for several different object beams, including OAM modes, their superpositions, and a few low‐order HG modes. For these measurements, we set the power of the reference and object beams to saturate the nonlinear response of ITO (both at $300\;GW/cm^{2}$, which is well below the damage threshold of roughly $2\;TW/cm^{2}$), and also ensure that time delays between object, reference, and read beams are optimal. We then determine the efficiency by measuring the ratio between input read beam and resulting image beam through power meter measurements. The efficiencies are summarized in Figure 3a, showing that the efficiency is maximal (≈3%) for a Gaussian object beam (ℓ=0) and decreases with an increase in the radius (|ℓ|>0) of the object beam. Because the fringe visibility of the induced metastructure depends on the modal overlap of the object and reference beams and the diffraction efficiency of the read beam depends on its overlap with the metastructure, the maximum efficiency is obtained when all three incident beams have the same mode shapes. In our implementation these conditions are achieved for an ℓ=0 object beam, since then all three beams have Gaussian shapes. As a consequence of the reduced modal overlap between the three beams, object beams carrying higher‐order OAM values (|ℓ|>0) or HG beams show lower efficiencies. Although even the lowest efficiency of a few percent shown here is as good as holograms made from photo‐refractive compounds [42], we also note that the efficiency can be greatly increased through appropriate nano‐structuring [43].

To gain insight into the intensity‐dependent formation property of our holograms, we measure the efficiency of an ℓ=+1 object beam by varying the power of the object beam while keeping the power of the reference Gaussian beam fixed at $300\;GW/cm^{2}$ (Figure 3b). We find that efficiency increases as a function of the intensity of the object beam reaching a near‐saturation value of approximately 3%. The contrast of the induced hologram, and consequently the efficiency of the information transduction depends not only on the beam parameters but also on the intrinsic linear and the nonlinear properties of the ITO layer and the relative vectorial orientation of the interacting beams with respect to the ITO film. In general, an interference pattern of an object and the reference beam may induce three different but spatially overlapped patterns: (1) a phase grating due to modulation of the real part of the index; (2) an absorption grating due to modulation of the absorption (i.e., the imaginary part of the index); and (3) an amplitude grating due to strong modulation of the Fresnel reflection coefficients. A strong modification of the Fresnel reflection is expected, because the real part of the index of ITO can change from a value of 0.4 to nearly 1.2 [29] resulting in strong angle‐dependent linear Fresnel reflectance and a large nonlinear modulation of the reflectivity. Consequently, the measured efficiencies for various object beams are impacted by the modulation of the complex optical constants of the ITO film and, thus, limited by the intrinsic saturation mechanisms of ITO's nonlinear response. For comparison, we note that the maximum diffraction efficiency of phase‐only sinusoidal gratings operating in the Raman–Nath diffraction regime (thin hologram) is 33.9% [44]. In our case the large modulation of the real part of the index (≈0.7) [29] can lead to a theoretical maximum diffraction efficiency of 22%. However, these high values of the maximum diffraction efficiency are drastically reduced as a result of the superimposed amplitude and absorption gratings over the phase gratings.

Having established experimentally that an ITO thin film can be used to transfer information between beams of light, we consider how its ultrafast nonlinear optical response [29] can also offer on‐the‐fly reconfigurability and rewritability using structured light fields. Figure 3c demonstrates this ultrafast behavior by the recorded efficiency of the holographic reconstruction while adjusting the delay between the formation of the hologram (i.e., the simultaneous arrival of the object and reference beams) and the read beam. We find that the non‐zero efficiency sustains over a few hundred femtoseconds of delay before the index distribution returns to the unstructured initial state. This effect implies that ITO could accommodate refresh rates up to 1THz, that is, up to nine orders of magnitude higher compared to what can be obtained using any traditional photo‐refractive material or polymers [45]. Although single light fields rarely reach such high rates of modulations, the achievable refresh rates will enable various applications such as its simultaneous use for multiple object beams with minimal cross‐talk, ultrafast temporal filtering, or its potential utilization in fields such as ultrafast transient holographic microscopy [46].

Although the light‐induced changes to the refractive index of ITO are the strongest near the zero‐permittivity wavelength, we are not limited to using read beams at the same wavelength as the reference and object beams. Measuring the maximum diffraction efficiency (ℓ=0) as a function of the read beam wavelength while keeping the reference and object beam fixed at 1260 nm, we find that a maximum absolute diffraction efficiency of 1% or more can be obtained in a wavelength range of more than 300 nm (Figure 3d). We note that for wavelengths larger than the zero‐permittivity wavelength, ITO becomes metal‐like. Consequently, for wavelengths larger than 1260 nm, the Fresnel reflectivity and absorption increases (i.e., the net transmission decreases), whereas the phase grating becomes weaker. We also note that it is not imperative that the reference and object beam wavelengths be kept at precisely 1260 nm. As long as they are within the above‐mentioned wavelength range, the resulting large contrast between the base index and nonlinearly modulated index enable a reasonable diffraction efficiency of the read beam into the image beams. Nevertheless, the large operating bandwidth suggests the suitability of ITO information transfer from one color of light to another for applications in wavelength‐division‐multiplexing schemes.

## 4 Demonstrating All‐Optical Operations

**FIGURE 4:**
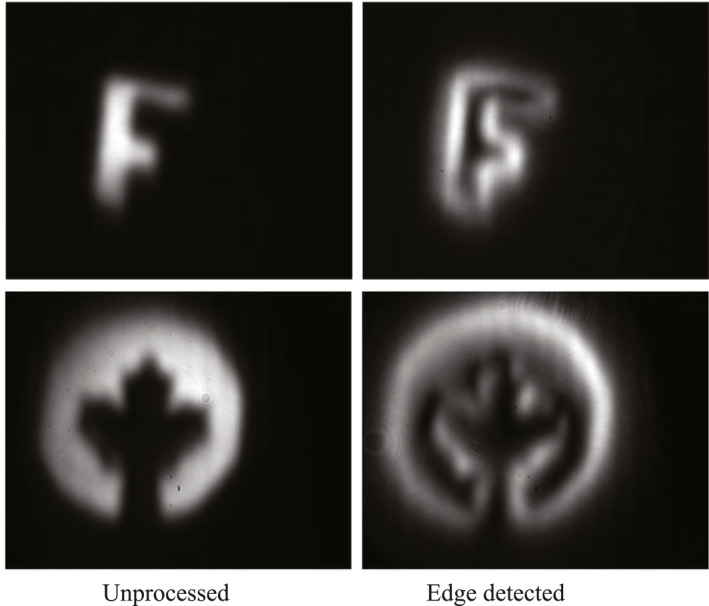
Holographic 2D spatial first‐order differentiation for edge detection. When the object beam carries a topological charge (ℓ=+1), the two‐dimensional spatial differentiation of the image‐carrying read beam is generated in the diffracted image beam.

The strong and ultrafast response of ITO not only enables holography far beyond video rates but also underlines that ITO is an ideal candidate for all‐optical analog computation using transversely structured light fields. The general idea would be to use the read beam as an input to a computation device whose operation is configured by the transformation kernel based on the complex scattering properties of the metastructure and induced by custom‐tailored object and reference beams. The image beam is the output of the operation. To demonstrate the potential of our approach for such computations, we implement an edge‐detection procedure for images carried by the read beam. We set the reference beam to be a Gaussian beam, whereas the object beam carries an OAM beam with a topological charge of ℓ=+1. As can be seen in the inset of Figure 1a, the interference structure of the reference and object beams induces a holographic metasurface with a fork grating in the ITO layer with a single dislocation. By diffracting an image carried by the read beam off such a fork grating corresponds to performing a generalized Hilbert transform or convolution tasks with special radially symmetric kernels [47]. Because the transform at any point depends on the spatial structure of the whole grating, the transform is nonlocal in nature and can also be understood as a first‐order optical differentiation operation [48]. In fact, the order of the spatial differentiation can be controlled by the topological charge of the object beam. In our experiment, we input two different images (amplitude structures) encoded in the read beam (Figure 4, left column). After undergoing a differentiation operation by the hologram, the resulting outputs are edge‐detected images (Figure 4, right column). The output images show a near‐uniform circularly symmetric response of our optical system, highlighting the isotropic response ITO and the ability to process complex images. We note that although the response of the material is dominated by the hot‐electron effect, we have not observed any discernible degradation of the image quality during our experiments. This is due to short pulses that we have used and the slow nature of all relevant diffusive processes. The example operations showcase the potential of our technique, which could be readily extended to more sophisticated holographic metastructures generated by multibeam interference and operations that could exploit both the image and conjugate image outputs. The versatility and rapid rewritability could enable complex computation tasks, including higher‐order differentiation, integration, and equation solving [49, 50].

## 5 Conclusions

In summary, we have presented a nonlinear optical approach to efficient, rapidly reconfigurable real‐time holographic protocols using a thin film of ITO. The demonstrated ENZ‐based material platform is capable not only of direct information transfer through the spatial amplitude and phase distributions of beams but can also perform all‐optical operations. The speed and efficiency with which this light‐encoded information can be read, modified and re‐written could make fast analog computation in scenarios where both time‐ and frequency‐division multiplexed operation are required.

In this report, we have demonstrated proof‐of‐principle implementations of real‐time holography using a nanomaterial. There are significant opportunities in improving the performance, including the energy expenditure, and functionality by orders of magnitude through judicious engineering. Given that the diffraction efficiency in our system is achieved by a combination of phase, absorption, and amplitude gratings, due to simultaneous modification of the real and the imaginary parts of the refractive index, we argue that manipulating the relative impact of each could boost the diffraction efficiency. Furthermore, the optimization of the linear optical properties and the dimensions of ENZ material, addition of plasmonic or dielectric structures, and implementation in reflection rather than transmission may also lead to increased efficiency although simultaneously decreasing the energy expenditure. Beyond the first‐order differentiation operation shown here and the ready extension to higher‐order differentiation, integration, and convolution operations, the nonlocal transformation could enable all‐optical re‐writable layers within computational neural networks, accelerate inverse design and prototyping systems, and facilitate adaptivity in multimode computation and imaging protocols. Consider, for example, an information‐carrying beam experiencing spatio‐temporal fluctuations from turbulence; a rapidly reconfigurable holographic surface could enable the recording and correction of disturbances through automatic phase conjugation in the diffraction process or through time gating. Finally, although the current scheme requires no nanofabrication, the diffraction efficiency could be enhanced while decreasing the intensity requirements, by orders of magnitude, by incorporating resonant nanostructured materials on the ITO layer [43]. Beyond the boost in performance, inclusion of resonant plasmonic or silicon nanostructures could also allow polarization‐dependent amplification, processing of information encoded in the spin‐degree of freedom, and the transfer of information between spin and the angular momentum of light [51]. Lastly, future work may involve exploring the application of the presented method to recent approaches for temporal signal processing [52], ultrafast modulation in space and time [53], and reprogrammable real‐time metasurface for machine learning and nonlinear optics [54, 55].

## Conflicts of Interest

The authors declare no conflicts of interest.

## Supporting information


Supporting Information S1

## Data Availability

The data that support the findings of this study are available from the corresponding author upon reasonable request.
