# Supplementary material for: An Epsilon‐Near‐Zero‐Based Nonlinear Platform for Ultrafast Re‐Writable Holography
Source: Nanophotonics. 2026 Jan 25;15(2):e70016. doi: 10.1002/nap2.70016 (PMC12964989; doi:10.1002/nap2.70016)
Supplement: Supplementary file 1 — Supporting Information S1 [file NAP2-15-e70016-s001.pdf]

Ultrafast re-writable holography for optical information processing: supplemental document

M. Zahirul Alam Robert Fickler Yiyu Zhou Enno Giese Jeremy Upham Robert W. Boyd

1 Material Properties

We purchased ITO films deposited on float glass substrates from Precision Glass & Optics (PG&O). We present the permittivity data of the material as obtained using ellipsometry in Fig. 1. The zero permittivity wavelength of the film is 1240 nm. The Drude parameters we used to model the permittivity of the material near the zero permittivity wavelength are $\epsilon_\infty = 3.77$, plasma frequency $\omega_p = 2.954 \times 10^{15}$ rad/s and damping coefficient $\Gamma = 0.0495\omega_p$.

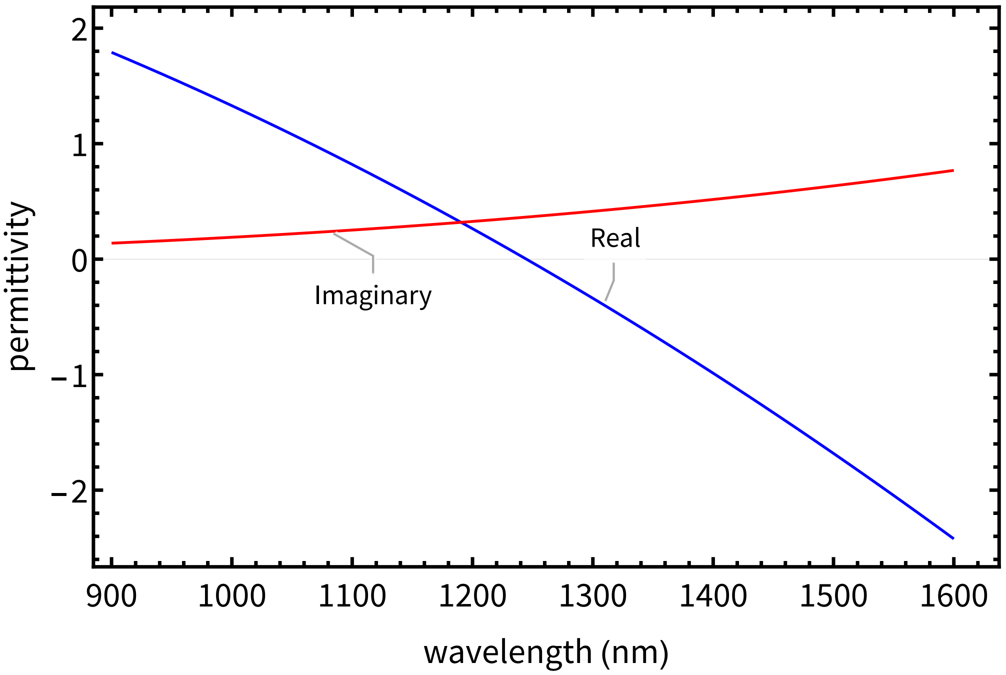

Figure 1: Permittivity of the ITO film used.

2 Experimental Setup

We use a pair of optical parametric amplifiers (OPA) pumped by a modelocked Ti:Saph laser producing 120-fs pulses at a wavelength of approximately 1260 nm and a repetition rate of 1 kHz. For both outputs, we remove residual light of a wavelength shorter than 1000 nm using a long-pass filter, and we clean the spatial mode through a pair of apertures. We use the beam of one OPA as the read beam such that we can adjust its wavelength separately. We split the beam of the other OPA using a thin-film beam splitter to obtain the reference and the object beams, see Fig. 2 . Using a half-wave plate followed by a Glan-Taylor polarizer in each of the three beams, we ensure that all beams are p-polarized and their respective intensities are independently controllable.
Additionally, we control the length of the beam paths of the read and the reference beam using a pair of motorized delay lines, such that we can ensure the simultaneous arrival of all three pulses on the ITO sample, and we can also perform the pump-probe experiment with controllable time delay as described in the main text. The object beam is structured using computer-generated holograms implemented by a liquid-crystal phase-only spatial light modulator. We use a Hamamatsu spatial light modulator displaying computer generated holograms to generate the spatial modes (see inset for an example of a hologram

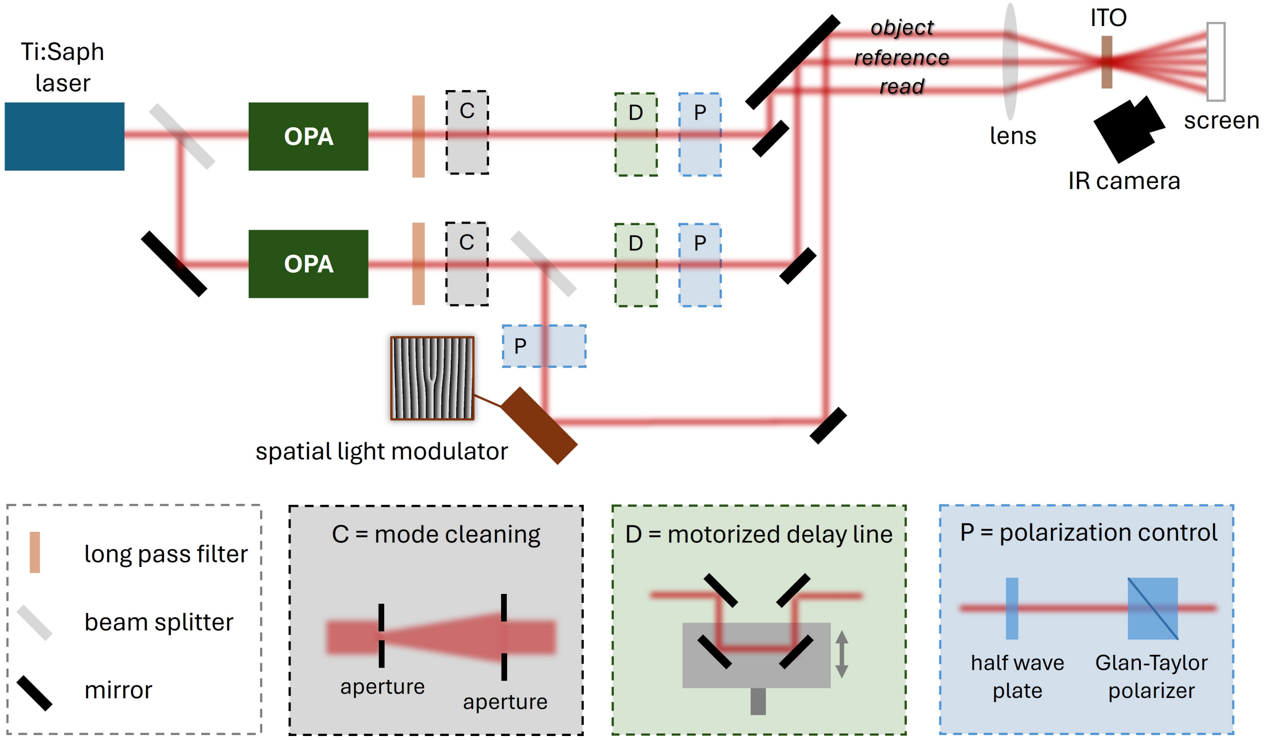

Figure 2: Simplified sketch of the experimental setup.

imprinting an OAM mode of $\ell = 1$). Although the SLM is not designed to be used at wavelengths of interest for this work, the SLM is functional at these wavelengths but with a a slightly reduced diffraction efficiency. We used Matlab to generate the holograms for the spatial modes. The letter "F" was made by cutting a thin anodized aluminum sheet. The maple leaf structure was a metal mask made using a commercial laser cutting service. Finally, we focus all three beams jointly onto the ITO using a single two-inch lens and record all beams, including the newly generated image beams, using a white screen, which we image by an IR camera. For all efficiency measurements given in the main text, we used the screen and camera only to simplify the alignment but recorded all power values by putting a power meter in the required beam path (not shown in Fig. 2).

3 Unprocessed camera recording

In Fig. 3(a) we present the unprocessed version of the camera recording shown in Fig. 1 in the main text. In the main text we adjust two regions where the two beams, image and conjugate beam, are brightened by a factor of 10 for better visibility. Using a different colormap, the image and image conjugate beams become better visible without the need to increase the brightness of the signal and conjugate beams, see Fig. 3(b).

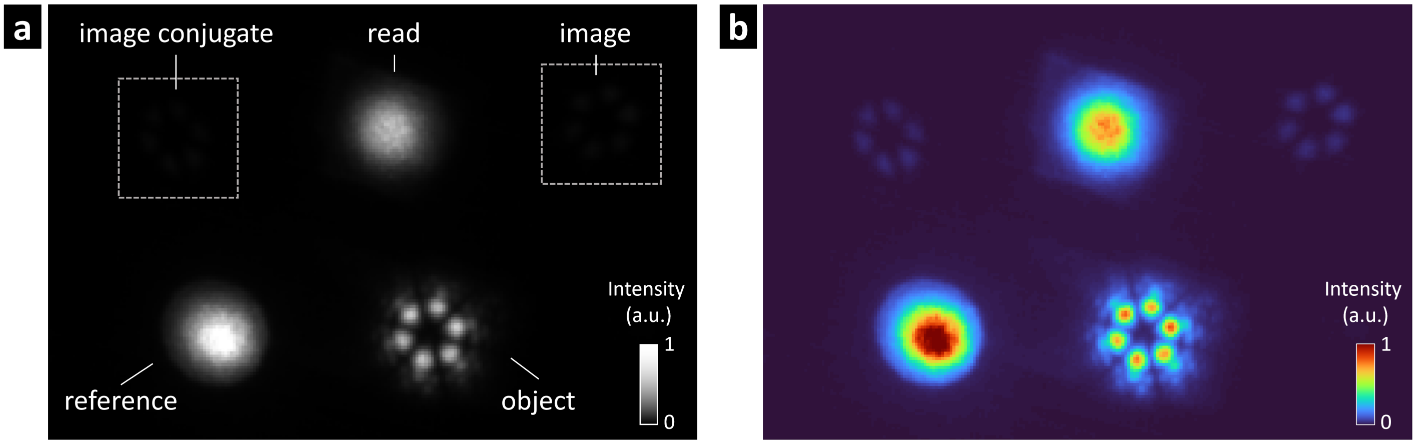

Figure 3: Original recording of all beams involved in the experiment. a) The unprocessed image shows all beams, object, reference, read, image, and image conjugate as labeled in the main text. However, the image and image conjugate are barely visible because of the low dynamic range of the camera. b) Using a different colormap (see inset), both image beams become better visible, without the need to brighten the regions.
